# A consensus multi-view multi-objective gene selection approach for improved sample classification

**DOI:** 10.1186/s12859-020-03681-5

**Published:** 2020-09-17

**Authors:** Sudipta Acharya, Laizhong Cui, Yi Pan

**Affiliations:** 1 Big Data Institute, College of Computer Science and Software Engineering, Shenzhen University, Shenzhen, PR China; 2 Department of Computer Science, Georgia State University, Atlanta, USA

**Keywords:** Feature selection, Sample classification, Gene ontology (GO), Protein protein interaction network, Multi-view clustering, Multi-objective optimization

## Abstract

**Background:**

In the field of computational biology, analyzing complex data helps to extract relevant biological information. Sample classification of gene expression data is one such popular bio-data analysis technique. However, the presence of a large number of irrelevant/redundant genes in expression data makes a sample classification algorithm working inefficiently. Feature selection is one such high-dimensionality reduction technique that helps to maximize the effectiveness of any sample classification algorithm. Recent advances in biotechnology have improved the biological data to include multi-modal or multiple views. Different *‘omics’* resources capture various equally important biological properties of entities. However, most of the existing feature selection methodologies are biased towards considering only one out of multiple biological resources. Consequently, some crucial aspects of available biological knowledge may get ignored, which could further improve feature selection efficiency.

**Results:**

In this present work, we have proposed a Consensus Multi-View Multi-objective Clustering-based feature selection algorithm called **CMVMC**. Three controlled genomic and proteomic resources like gene expression, Gene Ontology (GO), and protein-protein interaction network (PPIN) are utilized to build two independent views. The concept of multi-objective consensus clustering has been applied within our proposed gene selection method to satisfy both incorporated views. Gene expression data sets of *Multiple tissues* and *Yeast* from two different organisms (*Homo Sapiens* and *Saccharomyces cerevisiae*, respectively) are chosen for experimental purposes. As the end-product of CMVMC, a reduced set of relevant and non-redundant genes are found for each chosen data set. These genes finally participate in an effective sample classification.

**Conclusions:**

The experimental study on chosen data sets shows that our proposed feature-selection method improves the sample classification accuracy and reduces the gene-space up to a significant level. In the case of *Multiple Tissues* data set, CMVMC reduces the number of genes (features) from 5565 to 41, with 92.73% of sample classification accuracy. For *Yeast* data set, the number of genes got reduced to 10 from 2884, with 95.84% sample classification accuracy. Two internal cluster validity indices - Silhouette and Davies-Bouldin (DB) and one external validity index Classification Accuracy (CA) are chosen for comparative study. Reported results are further validated through well-known biological significance test and visualization tool.

## Background

The high dimensionality of gene expression data set causes the data-analysis techniques working inefficiently. Sample classification of gene expression data is one such data-analysis method that is majorly affected by large number of genes present within a data set. As a solution, in the past several years, researchers have come up with dimensionality reduction methods following different strategies [1–4]. It can be done in two ways; 1) Feature extraction: which combines different available features and creates a new feature and, 2) Feature selection: which eliminates irrelevant features and keeps a smaller subset of available features. In the current article, we are focused on developing an efficient feature selection methodology. Here, genes of expression data sets are treated as features; hence, throughout the paper, we will use the term ‘gene selection’ and ‘feature selection’ alternatively.

In gene expression data, tissue samples are associated with expression values of thousands of genes. But in reality, only a smaller subset of genes actively participates in sample classification. Therefore, it is necessary to eliminate irrelevant genes in order to 1) make data more interpretable 2) reduce the noise, which intuitively improves classification accuracy, and lastly, 3) decrease computational cost. An efficient gene selection algorithm is capable of reducing the gene-space dimension of a expression data set without losing relevant biological information. This embedded relevant biological data is essential for sample classification.

The existing feature selection algorithms can be based on different methods such as clustering-based [3, 5], graph-based [6], feature similarity-based [7], content-based [8]. The traditional clustering-based feature selection methods [3, 5] often suffer from inter-method inconsistency in assigning data points to respective clusters [9]. Therefore, obtaining consensus clusters of multiple clustering solutions can improve the overall feature selection performance.

Another point of our motivation is that biological data can be interpreted in different ways. In other word, biological data is ‘multi-omics’ (such as genomics, proteomics, and methylomics) or having multiple ‘views’ ([10–12]). For example, to identify clusters of ‘similar’ genes, several biological resources like gene expression data, gene annotation data from GO, protein interaction data from PPIN, protein sequence data can be used independently. Each of these controlled vocabularies interprets gene-gene similarity in different aspects or views. Therefore, each of them produces different clustering solutions. Single-omic clustering has proven invaluable for non-biological, biological and medical research [13, 14]. Hence, for the problem mentioned above, the consensus of different views certainly helps to achieve better clustering solutions of similar genes. Though in recent years, several clustering-based gene selection strategies have been developed to identify the non-redundant set of genes, most of them are single ‘view’ or ‘omics’ data-based. In articles like Acharya et al. [5], Mitra et al. [3], the gene selection task is solved through single-view clustering approaches.

### Related works and motivation

In the last few years, several efficient feature/gene selection algorithms have been proposed. Some existing works relevant to this paper is discussed here in brief. A supervised feature selection technique was proposed by Liu et al. [15], which combines recursive feature addition (RFA) - a supervised learning method and a statistical similarity measure. Their proposed method considers only gene expression data. In contrast, Acharya et al. [5] proposed an unsupervised gene selection method based on annotation data of GO. Their method does not consider gene expression data but only GO annotation data. A single-objective genetic algorithm-based feature selection method can be found in Gunavathi et al. [2]. For sample classification utilizing their obtained feature set, kNN and support vector machine (SVM) classifiers are used. In Mirta et al. [3], authors developed a gene selection algorithm named Clustering Large Applications based upon RANdomized Search (CLARANS), using GO annotation data and then performed single-objective clustering on samples of gene expression data. A graph-theoretic approach of feature selection was proposed by Mandal et al. [6], where, from input gene expression data, a weighted dissimilarity graph is created. The nodes of the graph represent genes and edges represent their dissimilarity. More of the edge weight represents the more dissimilarity, and higher node weight represents the corresponding gene is highly relevant. Finally, the feature selection problem is modeled as a dense sub-graph finding problem that is solved through multi-objective binary particle swarm optimization (MBPSO).

One point common about the above-mentioned existing works is that all of them are single-view approaches. The working principles of most of the methods are either only based on gene expression data or gene annotation data from GO.

Recently, applications of multi-view learning on feature selection has become very popular. But, most of existing multi-view feature seletion methods are designed for video or text data analysis. For example, in Xu et al. [16], authors have proposed a weighted multi-view clustering and feature selection technique and shown its application on real-life text and image data sets. Also, their proposed approach follows single-objective clustering methodology. Similarly, in Shao et al. [17], an online unsupervised multi-view feature selection algorithm has been proposed by authors, but that is also for video or text data analysis. In Liu et al. [18] and Xue et al. [19], authors have developed feature selection algorithms using multi view data for video, text, and image data sets. On the problem of gene selection, an unsupervised graph-theoretic multi-view clustering approach was proposed bt Swarnkar et al. [20]. Though their proposed algorithm is a multi-view approach, here, different ‘views’ are developed by considering different feature subspaces over a gene expression data set. So, multi-omics data has not been utilized by them. A good review article on available multi-view feature selection algorithms can be found in Zhang et al. [21] and Yang et al. [22].

From the existing literature, it is evident that limited work has been done on the development of multi-view feature selection methods for biological data. Hence, there is a significant scope to explore this area. Inspired by this, in the current article, we have proposed an unsupervised consensus multi-view gene selection approach called CMVMC (Consensus Multi-View Multi-objective Clustering). we have amalgamated three concepts in our proposed gene selection method, such as consensus clustering, multi-view learning, and multi-objective optimization. Three primary controlled genomic/proteomic sources, i.e., gene expression, GO, and PPIN are chosen to form two different views. To the best of our knowledge, our proposed multi-view multi-objective approach of gene selection is new in this problem domain and has not explored before.

## Methods

In this section, first, we have formulated the chosen problem mathematically. Next, we have described the building mechanism of two views for the proposed multi-view framework. Finally, we will describe different steps of CMVMC-based gene selection approach.

### The mathematical formulation of chosen problem

- Given:
  - An original gene expression data set of *n* genes *G*_*org*_={*g*_1_,*g*_2_,…,*g*_*n*_}.
  - Each gene has expression values of *d* different samples.
  - Total *v* different views, i.e., *v* number of *n*×*n* similarity matrices.
  - (*v*+1) number of objective functions
    $$OF_{1}, OF_{2},\ldots, OF_{v}, AI, $$
    where *OF*_*i*_ is a fitness function of the clustering solution corresponding to view *i*. The last objective function is Agreement Index (*AI*), which measures the agreement between clustering solutions obtained by *v* views.
- Outcomes:
  - A consensus clustering solution (*CU*) of similar genes, which satisfies *v* views and simultaneously optimizes (*v*+1) objective functions.
  - The genes of *G*_*org*_, is partitioned into *K* clusters, {*CU*_1_,*CU*_2_,…,*CU*_*K*_}
  - $CU_{i}=\left \{{g}^{i}_{1}, {g}^{i}_{2}, \ldots, {g}^{i}_{n_{i}}\right \}$; *n*_*i*_: number of points in cluster *i*; ${g}^{i}_{j}$: *j*^*th*^ point of consensus cluster *i*.
  - $\cup _{i=1}^{K} CU_{i} =G_{org} $ and *CU*_*i*_∩*CU*_*j*_=*∅* for all *i*≠*j*.
  - *K* cluster medoids of consensus clustering solution, which can be declared as selected features (genes) after proper validation test.
  - A reduced gene expression data set with *K* genes *G*_*org*_={*g*_1_,*g*_2_,…,*g*_*K*_}.

### Two designed views of CMVMC

**Fig. 1:**
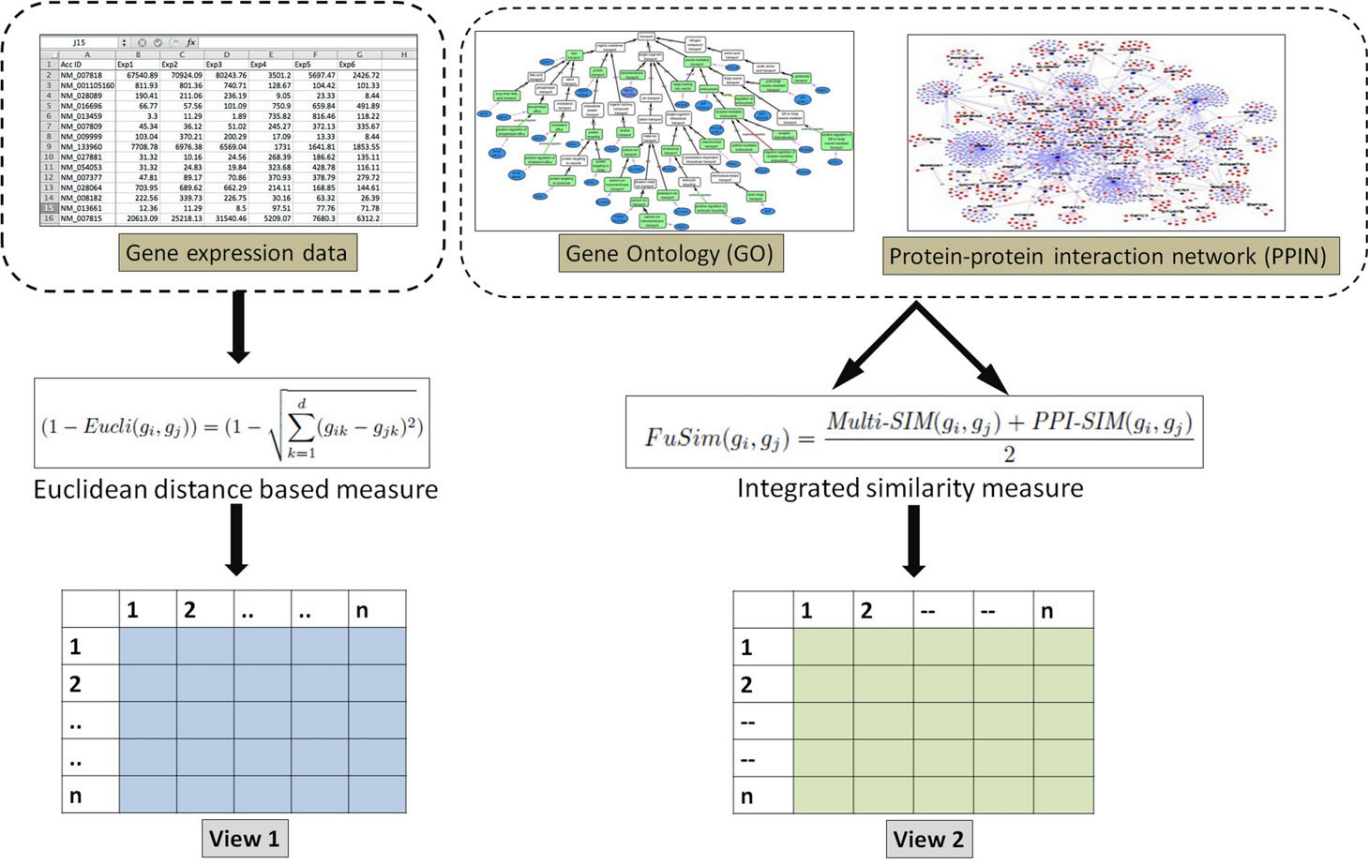
Two views developed based on multiple *‘omics’* data

The first view used in CMVMC is the gene-gene similarity network based on pair-wise Euclidean distance between expression vectors of genes. It represents the similarity between genes based on their sample-specific expression values. For the second view, the gene-gene similarity network is created utilizing a very recently developed fused gene-gene similarity measure *FuSim* [23]. This measure incorporates the biological properties of genes, both from GO and the corresponding organism’s PPIN. The promising performance of *FuSim* inspired us to choose it for defining the second view. The similarity network of this view signifies the semantic and functional similarity between genes, which is not specific to samples but captures a global functional relatedness among them. The formation of both views is illustrated graphically in Fig. 1.***Building view 1:***Let us consider *n* = number of genes and *d*= number of samples in the input gene expression data set. The original expression data is represented as *G*_*org*_[*n*][*d*]. *g*_*ik*_ represents expression value of *i*^*th*^ gene of data set for *k*^*th*^ sample where, *i*∈*n* and *k*∈*d*. The similarity network for *view 1* is represented as two-dimensional similarity matrix *S*_*View*1_[*n*][*n*] of dimension *n*×*n*. Each entry of *S*_*View*1_[][] matrix is calculated according to Eq. 1.

1 $$ \left(1 - Eucli(g_{i},g_{j})\right)=\left(1 - \sqrt{\sum_{k=1}^{d}(g_{ik}-g_{jk})^{2}}\right)  $$

where, *i*,*j*∈*n* and *i*≠*j*. We have chosen Euclidean distance as it is widely accepted and chosen distance measure while identifying co-expressed genes. The *Eucli*(*g*_*i*_,*g*_*j*_) is subtracted from 1, which indicates the pair-wise similarity between gene *g*_*i*_ and *g*_*j*_. ***Building view 2:***For *view 2*, the corresponding similarity matrix *S*_*View*2_[*n*][*n*] is created by calculating pair-wise similarity measure as follows.

2 $$ FuSim \left(g_{i}, g_{j}\right) = \frac{\textit{Multi-SIM}(g_{i},g_{j}) + \textit{PPI-SIM}\left(g_{i}, g_{j}\right)}{2}  $$

where, *Fusim*(*g*_*i*_,*g*_*j*_) is GO (Acharya et al. [24]) and PPIN based hybrid similarity measure between gene *g*_*i*_ and *g*_*j*_ according to Acharya et al. [23]. *Multi*-*SIM*(*g*_*i*_,*g*_*j*_) is GO-based multi-factored measure and *PPI*-*SIM*(*g*_*i*_,*g*_*j*_) is confidence of association within PPI-based similarity measure between genes. The detailed mathematical formulation of Eq. 2 can be found in Acharya et al. [23].

The developed two views i.e., *S*_*View*1_[*n*][*n*] and *S*_*View*2_[*n*][*n*] are employed as different views in proposed CMVMC-based gene selection algorithm.

### Working strategy of proposed CMVMC-based gene selection method

**Fig. 2:**
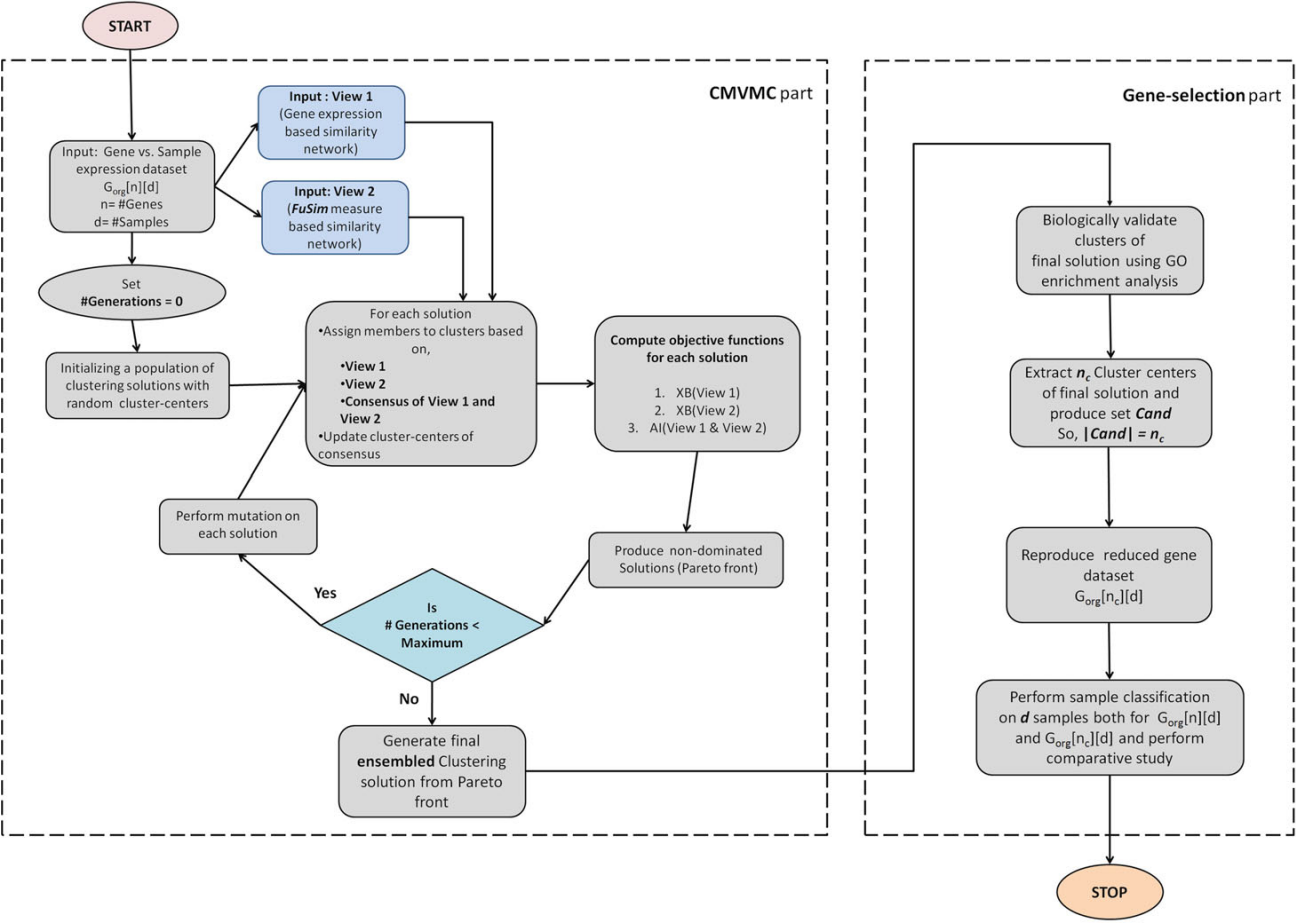
The flowchart of proposed CMVMC-based gene selection algorithm

In this section, each step of CMVMC-based gene selection algorithm is described in detail. The working flowchart of proposed gene selection method is shown in Fig. 2.

#### Step 1: initializing clustering solutions

**Fig. 3:**
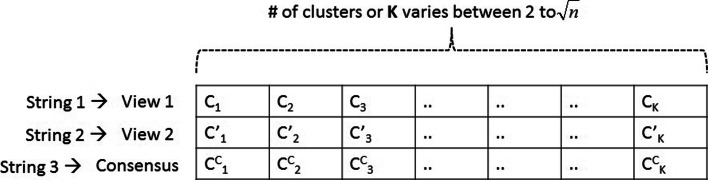
Structure of each *parent* clustering solution in proposed CMVMC

The execution of CMVMC starts with the initialization of a number of clustering solutions with randomly selected centroid genes from the set of ‘*n*’ genes in a given data set. The structure of a single solution is shown in Fig. 3. We can see from the figure that both for *view 1* and *view 2*, two separate strings (String 1 and String 2) within a single solution are created. String 3 within the same solution represents the consensus of String 1(*view 1*) and String 2(*view 2*). We called the clustering solution containing all three strings a *‘Parent’* solution. String 3 is updated in each iteration (except the first iteration, initialized as null string). Each string (String 1, String 2, and String 3) of *parent* solution represents a complete clustering solution developed following its corresponding view. As shown in Fig. 3, C _*i*_ and C’ _*i*_ are cluster center or centroid genes of *i*^*th*^ cluster corresponding to *view 1* and *view 2*, respectively. C$^{c}_{i}$ is the *i*^*th*^ consensus-cluster center of String 3. *K* denotes the number of clusters. CMVMC detects the number of clusters in an optimized solution automatically; hence, *K* varies between range 2 to $\sqrt n$ [25] for each clustering solution.

#### Step 2: assigning genes to clusters and creating consensus clustering solution

In this step, the rest of the genes (except centroid genes) are assigned to corresponding clusters of both strings (String 1 and String 2) within a *parent* solution. For a particular string, a gene is assigned to a cluster whose centroid gene is most similar to that gene concerning the corresponding view. The similarity is measured utilizing associated similarity matrices - *S*_*View*1_[*n*][*n*] and *S*_*View*2_[*n*][*n*] as developed previously.

**Fig. 4:**
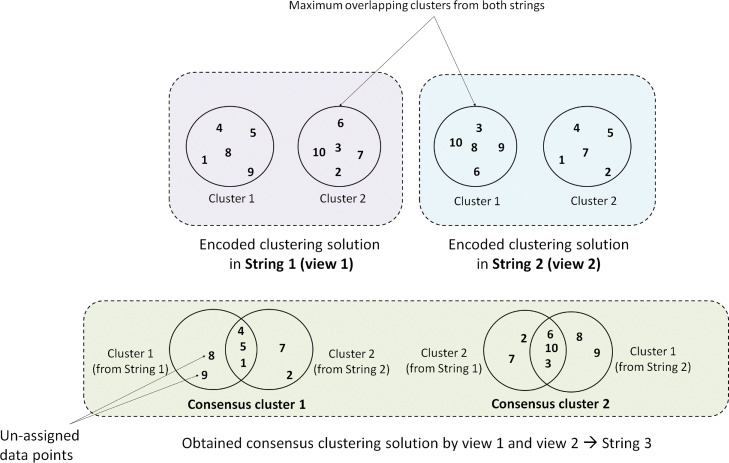
Formation of consensus clusters of *view 1* and *view 2*

Once individually, both strings are full of assigned genes, the consensus clustering solution combining String 1 and String 2 is created. The complete consensus solution is represented by String 3. An example of our strategy of consensus clustering is graphically illustrated in Fig. 4. First of all, the maximum overlapping clusters between String 1 and String 2 are identified. Next, the most centrally located gene among common genes between two mostly overlapping clusters is chosen as the new center of consensus cluster. The rest genes which can not be categorized are assigned to consensus clusters according to maximum-average-similarity strategy utilizing both *S*_*View*1_[*n*][*n*] and *S*_*View*2_[*n*][*n*].

#### Step 3: measuring objective functions

CMVMC is a multi-objective clustering approach; hence, like other multi-objective optimization algorithms, it optimizes multiple fitness functions at its every iteration. For each *parent* solution, three of our chosen objective functions are Xie Beni (XB) index [26] for String 1, XB index for String 2, and Agreement Index (AI) [27] between String 1 and String 2. XB index measures cluster quality concerning better intra-cluster and inter-cluster distance and have been chosen in several past works of literature in recent years [23, 28]. For a good clustering solution, the XB index value should be minimum.

The third objective function AI [27] represents the similarity between clustering solutions obtained by two String 1 and String 2 (following both views) and higher its value signifies a better solution. The formulation of AI between two clustering solutions *CS*_*view*1_ and *CS*_*view*2_ based on two views, *view1* and *view2*, is described as follows.

Assume the agreement matrices corresponding to clustering solution *CS*_*view*1_ and *CS*_*view*2_ are *AM*^*view*1^ and *AM*^*view*2^ respectively. If there are *n* genes, then the dimension of each agreement matrix is (n × n). From both obtained agreement matrices, the count of agreements (*AG*) is *AG*= ∑*i*=1*n* ∑*j*=1*nI*_*AMijview*1,*AMijview*2_ where,

$$\begin{array}{@{}rcl@{}} I_{AM_{ij}^{view1}, AM_{ij}^{view2}}&=& 1 ~~~\text{if}~~AM_{ij}^{view1}= AM_{ij}^{view2}\\ &=&0~~~~\text{otherwise} \end{array} $$

Also the count of disagreements (*DG*) is *DG*=*n*^2^−*AG*. Hence the AI between two clustering solutions *CS*_*view*1_ and *CS*_*view*2_ is formulated as follows.

$$AI_{CS_{view1},CS_{view2}}=\frac{AG+1}{DG +1} $$

To avoid the *division by zero* problem, ‘1’ is added as a normalization factor.

As the underlying multi-objective optimization strategy and generating non-dominated solutions in each iteration, we have used Archived Multi-Objective Simulated Annealing (AMOSA) [29] within CMVMC. In the field of multi-objective optimization, AMOSA has shown its superiority over several other multi-objective as well as single-objective optimization techniques. Its unique characteristic of accepting bad solution makes AMOSA not-to-get-stuck into local optima and hence provide optimal solutions.

#### Step 4: perturbation operators

To achieve optimal clustering solution and hence to explore the search space efficiently, three types of perturbation operators are applied on both strings (String 1 and String 2) of each *parent* solution. They are described in brief as follows.**Perturbation 1:** This perturbation operator aims to replace one existing centroid-gene from String 1 and String 2 with another gene. The gene to be selected as a new centroid is drawn from the original dataset randomly. Then it is replaced with an existing centroid-gene from a random position of the string.**Perturbation 2:** This operation aims to increase the number of encoded clusters both in String 1 and 2 by one. For each string, a random gene from the original data set is chosen and added as the centroid of a new cluster.**Perturbation 3:** This is the last type of perturbation operator, which aims to decrease the number of clusters by one from each string. A random position from both strings is chosen and assigned with a null value. Hence, the clusters count is decreased by one.

After performing any of the three mentioned mutation types, *Step 2* is repeated as membership has to be re-computed for the new cluster centers as well as consensus partitioning.

#### Step 5: choosing the final clustering solution through ensemble clustering

In the end, CMVMC produces multiple non-dominated Pareto optimal clustering solutions [30]. Next, all of the consensus partitions obtained from different non-dominated solutions are ensembled following the majority voting strategy. According to this strategy, if a pair of genes are members of the same cluster for the majority of produced consensus clustering solutions, then they kept together in the final solution. The rest unallocated genes are placed to corresponding clusters by following the maximum-average-similarity (average similarity based on *S*_*View*1_[*n*][*n*] and *S*_*View*2_[*n*][*n*]) strategy as mentioned in *Step 2*. The ensembled clustering solution is further utilized to identify the set of relevant candidate genes.

#### Step 6: validate the obtained final clustering solution and prepare reduced gene set

Before obtaining the candidate genes from the ensembled clustering solution, to ensure that the obtained solution is biologically validated, we have performed GO enrichment analysis. GO tool (http://geneontology.org/) has been used for this significance test. After validating the clustering solution, the centers of obtained clusters embedded within the solution are extracted as candidate genes (features) of reduced gene-space. Suppose, this set of candidate genes is represented as *Cand*. Let, |*Cand*|= *n*_*c*_, which represents *n*_*c*_ number of genes are selected as relevant features/genes. Here, *n*_*c*_<*n* and also *n*_*c*_ = # of clusters in the final solution. Next, from the original expression data set - *G*_*org*_[*n*][*d*], rows corresponding to *n*_*c*_ candidate genes are only kept in reduced expression data set - *G*_*org*_[*n*_*c*_][*d*]. This is the end product of CMVMC-based gene selection algorithm. Finally, the sample classification on *d* samples can be performed for obtained gene expression data set with reduced dimensionality.

## Results

### Description of chosen data sets and specifications of views

For this study, we have chosen benchmark gene expression data sets of *Multiple tissues* (http://portals.broadinstitute.org/cgi-bin/cancer/datasets.cgi) and *Yeast* (http://arep.med.harvard.edu/). Also, their corresponding PPINs, i.e., *Homo Sapiens* (*H. Sapiens*) and *Saccharomyces cerevisiae* (*S. Cerevisiae*), respectively, are utilized. The PPINs are downloaded from HitPredict [31] - an open-access resource of experimentally determined protein-protein interaction data over several organisms. Originally, *Multiple tissues* gene expression data set has 5565 genes(features) and 103 samples. Similarly, *Yeast* has 2884 genes and 17 samples. 103 samples of *Multiple tissues* are distributed among four normal tissue types, which are prostate, breast, colon, and lung. Similarly, 17 samples of *Yeast* are devided in two major classes and again, each major class is divided into four sub-classes G1, S, G2, and M [32]. The specifications, of *view 1* and *view 2* for both data sets are also noted as follows.

- *Multiple tissues*: To generate *view 2*, we have first obtained annotated GO-terms of 5565 genes using the online tool Gene Ontology Resource (http://geneontology.org/docs/download-ontology/). The full GO-tree also downloaded from the same source. The number of mapped genes was 5205. The corresponding protein UniProt IDs of mapped 5205 genes are downloaded from ‘https://www.uniprot.org/uploadlists/’. The protein IDs are used to retrieve their interacting proteins and associated statistics from downloaded *H. Sapiens* PPIN. Once these data are ready, then according to Eq. 2, *FuSim* measure [23] is calculated between each pair of 5205 genes and the gene-gene similarity network *S*_*View*2_[][] of dimension (5205×5205) is created as *view 2*.
  To generate *view 1*, the sample vectors of the same 5205 mapped genes are extracted from the original gene expression data *G*_*org*_[*n*][*n*]. Then according to Eq. 1, the pair-wise similarity between all genes is calculated, and the similarity network *S*_*View*1_[][] of dimension (5205×5205) is obtained and treated as *view 1*.
- *Yeast*: The view generation procedure for this data set is the same as the previous data set. Out of 2884 genes, 2307 genes get mapped to one or more significant GO-terms by our chosen GO tool. Following the same strategy as mentioned before, the similarity networks or views *S*_*View*1_[][] and *S*_*View*2_[][] (of 2307×2307 dimension) are generated.

### Chosen external and internal validity measures

For that purpose of comparative analysis, two widely used internal cluster validity measures, which are Silhouette index [33] and DB index [34] have been used. A higher value of the Silhouette index and lower value of DB index indicates a better clustering solution. Also, to compare sample classification outcomes with original sample classes, an external validity index - Classification accuracy (CA) has been utilized.

## Discussion

### Discussion on results for *Multiple tissues* data

For *Multiple tissues* data set, at first, we have executed the CMVMC-based gene selection algorithm on its 5205 genes to identify gene clusters. To perform a comparative study, we have also executed single-view versions (considering *view 1* and *view 2* separately) of our proposed algorithm (Unsupervised Multi-objective Clustering (UMC)) on the chosen set of genes. We have also compared our obtained results with the approach proposed in Acharya et al. [5], which is shown in Table 1. From the table, we can see, for CMVMC, the final ensembled clustering solution has 41 clusters (indicated in bracket ‘()’ in Table 1), and the corresponding Silhouette value is 0.446. For the dimension of reduced gene-space, the method of Acharya et al. [5] attains a slightly better dimension, i.e., 40 genes. The aim of gene selection is not only to reduce the dimension of gene-space but also to select relevant and non-redundant genes. Consequently, a better clustering solution ensures to identify more relevant genes from its outcome. Our obtained solution is better in terms of Silhouette value compared to Acharya et al. [5] and other single-view approaches reported in Table 1. Hence, our obtained reduced gene-space with 41 genes seems more relevant.

**Table 1:** Silhouette values corresponding to optimal gene clustering solution obtained by proposed CMVMC as well as other clustering approaches. In bracket () number of gene clusters is mentioned

**Data sets**	**CMVMC**	**UMC-view1**	**UMC-view2**	**PAM(GO-based)**[5]
*Multiple tissues*	0.446(**41**)	0.434(**45**)	0.439(**47**)	0.4299(**40**)
*Yeast*	0.4671(**10**)	0.457(**13**)	0.462(**10**)	0.4531(**10**)

Next, we have performed the biological significance test through GO enrichment analysis (http://geneontology.org/) for the obtained clustering solution having 41 clusters. The outcome of this test for the random two clusters is shown in Table 2. In the table for each of the significant GO-terms, the percentage of genes from the obtained cluster and full genome set in GO tool sharing that term is reported. It is quite evident from the table that a higher percentage of genes from obtained clusters mapped into the corresponding GO-term compared to the full genome set. This outcome indicates that genes within a same cluster of obtained solution are more involved in similar biological processes compared to other genes in the data set.

**Table 2:** Biological significance test outcome (under ‘Biological process’ ontology) for random two gene clusters obtained by CMVMC for both data sets

*Multiple tissues*				*Yeast*			
**Cluster**	**Significant GO-term**	**Cluster %**	**Genome %**	**Cluster**	**Significant GO-term**	**Cluster %**	**Genome %**
Cluster 1	GO:0050896	55.38%	40.14%	Cluster 1	GO:0071704	61.2%	53.7%
134 genes	response to stimulus			217 genes	organic substance metabolic process		
	GO:0071840	40.7%	27.86%		GO:0044237	63.8%	54.8%
	cellular component organization or biogenesis				cellular metabolic process		
	GO:0048518	42.9%	29.3%		GO:0044249	31.4%	24.5%
	positive regulation of biological process				cellular biosynthetic process		
	GO:0032501	41.65%	33.07%		GO:0016043	43.4%	33.4
	multicellular organismal process				cellular component organization		
	GO:1901564	36.3%	25.3%		GO:0044260	42.8%	33.5%
	organonitrogen compound metabolic process				cellular macromolecule metabolic process		
Cluster 2	GO:0065008	37.39%	19.24%	Cluster 2	GO:0006807	53.9%	47.18%
124 genes	regulation of biological quality			197 genes	nitrogen compound metabolic process		
	GO:0051049	19.76%	8.8%		GO:0071840	40.09%	37.4%
	regulation of transport				cellular component organization or biogenesis		
	GO:0032879	26.85%	13.12%		GO:0016043	40%	33.4%
	regulation of localization				cellular component organization		
	GO:0009966	25.04%	14.89%		GO:0065007	41.7%	34%
	regulation of signal transduction				biological regulation		
	GO:0051128	24.05%	11.69%		GO:0043170	50.8%	40.4%
	regulation of cellular component organization				macromolecule metabolic process		

**Fig. 5:**
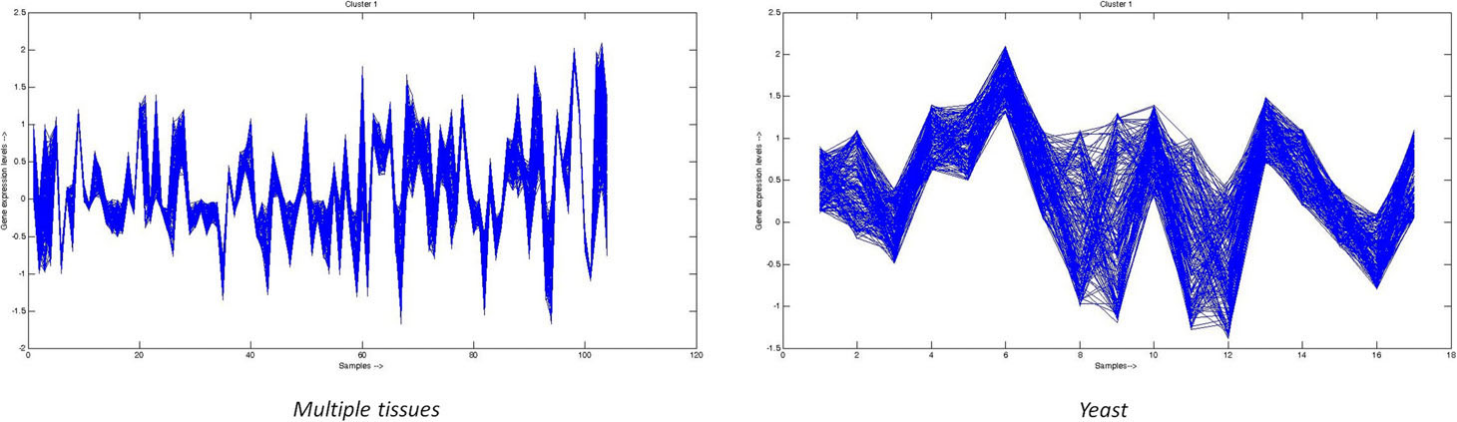
Cluster-profile plot for one random gene cluster from *Multiple tissues* (131 genes and 103 samples) and *Yeast* (180 genes and 17 samples) data set

Also, to visualize the quality of obtained clusters, we have plotted expression values of genes through cluster-profile plot corresponds to one random cluster out of 41 clusters. It is shown in Fig. 5. The x-axis of the plot represents samples, and the y-axis represents expression value of a gene corresponds to a sample. As we can see in Fig. 5, the expression values of genes of the obtained cluster are mostly co-aligned. It represents genes within the same cluster are co-expressed. Therefore, it is another evidence for obtained clusters of being good quality.

Once the biological and visual validity of the obtained clustering solution is proved, next, centroid genes of 41 clusters are extracted. The IDs of these 41 genes are TMEM1, ZFR, ASH2L, RGS3, SMARCC1, TAF1C, BZRP, MAPK1, STAC, SEC22L1, PTGER1, HIPK3, PSMD12, ORM1, TPM3, HOXC5, CCL22, HIST2H2AA, CACYBP, RPL10A, KIAA0980, CRSP9, EPHX1, NPIP, CORO2B, KIAA0350, MYLK, DC12, ST5, RECK, ISGF3G, CDKN1A, LRPAP1, M6PRBP1, KIAA0792, CSTF1, MSL3L1, PDLIM7, SEC11L1, TNFRSF25, ZNF629, These genes act as candidate genes in obtained reduced gene-space. For *Multiple tissues* data set, originally, *n*=5565 and reduced gene-space becomes |*Cand*|= *n*_*c*_=41.

Next, the classification of 103 samples is performed for both original (5565 genes) and reduced *Multiple tissues* gene expression data set. Besides our proposed one, we separately executed UMC-view1 and UMC-view2 based gene selection algorithm on the original data (5565 genes) and chosen common genes between two obtained reduced gene sets. Later, we perform sample classification with this common gene set (34 genes). We named the approach as UMC-v1-v2. Also, the reduced gene expression data set (40 genes) obtained by the approach of Acharya et al. [5] (we named it as PAM(GO)) has been chosen for comparative study. For original, as well as these three different reduced gene expression data sets, sample clustering is performed utilizing AMOSA-based multi-objective clustering algorithm.

**Fig. 6:**
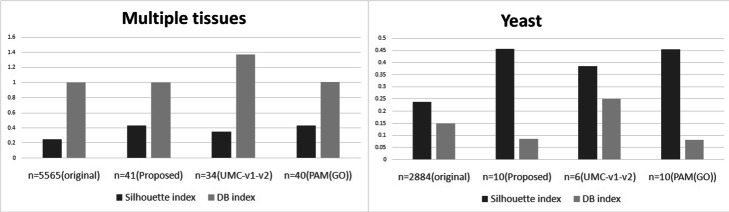
The comparative Silhouette and DB values for obtained sample clustering solutions for both data sets

**Fig. 7:**
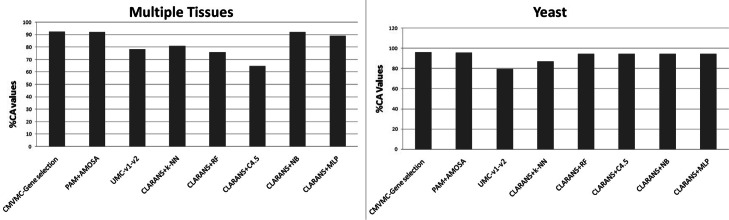
The comparative Classification Accuracy (CA) of samples by proposed and existing gene selection approaches

The output sample clustering solutions are compared with each other with respect to Silhouette and DB index. Corresponding results are reported in Table 3 and plotted in Fig. 6. From the reported results in Table 3, it is evident that sample clusters obtained from reduced data set (41 genes) by our method have better quality with respect to both indices. Again, the sample clustering outcome is compared with results reported in Acharya et al. [5] and Mitra et al. [3] with respect to classification accuracy (%CA). The obtained comparative result is shown in Table 4 and plotted in Fig. 7. From Table 4, it is evident that sample classification using the reduced gene set developed by CMVMC method meets higher accuracy compared to other gene selection strategies.

**Table 3:** Comparative analysis of obtained sample clusters with respect to internal validity measures

**Data sets**	**# of genes (features)**	**# of samples**	**Silhouette**	**DB**
*Multiple tissues*	5565 (original)	103	0.2527	0.998
	41(reduced by CMVMC)		**0.4314**	**0.997**
	34 (reduced by UMC-v1-v2)		0.3526	1.37
	40 (reduced by PAM(GO-based))		0.4299	1.0065
*Yeast*	2884 (original)	17	0.2365	0.149
	10 (reduced by CMVMC)		**0.4568**	0.087
	6 (reduced by UMC-v1-v2)		0.385	0.251
	10 (reduced by PAM(GO-based))		0.4531	**0.081**

**Table 4:** Comparative percentage of Classification Accuracy (%CA) values of proposed CMVMC-based gene selection as well as other existing methods

**Data set**	**# of genes**	**Algorithms**	**%CA**
*Multiple tissues*	**41**	CMVMC-gene selection	**92.73**
	40	PAM+AMOSA	92.14
	34	UMC-v1-v2	78.4
	42	CLARANS+k-NN	81.03
		CLARANS+RF	76.0
		CLARANS+C4.5	65.0
		CLARANS+NB	92.23
		CLARANS+MLP	89.32
*Yeast*	**10**	CMVMC-gene selection	**95.84**
	10	PAM+AMOSA	95.63
	6	UMC-v1-v2	79.5
	15	CLARANS+k-NN	86.78
		CLARANS+RF	94.12
		CLARANS+C4.5	94.12
		CLARANS+NB	94.12
		CLARANS+MLP	94.12

### Discussion on results for *Yeast* data

For *Yeast* data set, we have 2307 mapped genes on which we applied the proposed CMVMC-based gene selection technique and performed a comparative study with respect to other gene-selection algorithms. The results are reported in Table 1. Here, the reported Silhouette value corresponding CMVMC is better than the reported approach of Acharya et al. [5] by scale 0.014. According to our method, the number of genes in reduced gene-space is 10. The clustering solution obtained by UMC-view2 has Silhouette value 0.462, which is lesser than our proposed one, but it also detects a reduced set of 10 genes. For *Yeast* data set, *n*=2884 and the size of reduced gene-space becomes |*Cand*|= *n*_*c*_=10. The clusters of obtained clustering solution by CMVMC-based gene selection method are also validated through GO enrichment analysis. The outcome of the analysis is shown in Table 2. It is evident from the reported biological significance test outcome, that, obtained clusters are biologically enriched. Again, the cluster-profile plot for one random cluster from the final gene clustering solution, as shown in Fig. 5, shows that the genes within the same cluster are co-expressed. The IDs of 10 selected genes in the reduced gene-space are YBL084C, YGR152C, YOL058W, YDR379W, YDR165W, YLR325C, YFL008W, YLR103C, YPL150W, YOL047C.

Similar to *Multiple tissues*, with the original (2884 genes) as well as reduced gene expression *Yeast* data sets by different approaches, we have executed multi-objective clustering-based sample classification. The summarized results are shown in Table 3 an plotted in Fig. 6. Table 3 shows with respect to the Silhouette index, the quality of sample clusters obtained by CMVMC is better than other methods. However, it is slightly lower than the gene selection method of Acharya et al. [5] for DB index. The comparative classification accuracy of samples is reported in Table 4 and Fig. 7. The obtained results prove that the reduced gene-space produced by our method has more biologically informative genes compared to other methods. As a result, the sample classification with our obtained gene-space attains higher accuracy compared to others.

Finally, from the obtained results and comparative analysis, it is clear that CMVMC-based gene selection approach identifies the more informative, relevant, and non-redundant set of genes from both data sets, which also assists in attaining higher accuracy in sample classification.

## Conclusions

In the current work, we have developed an unsupervised consensus multi-view multi-objective gene selection approach (CMVMC-gene selection) for efficient sample classification. Multi-faceted biological sources like gene expression data, GO, and PPIN are involved in defining two views of gene-gene similarity networks. These complimentary views are further utilized by the proposed gene selection algorithm. Experiments are performed on two widely used benchmark gene expression data sets - *Yeast* and *Multiple tissues*. Their corresponding PPINs of *S. Cerevisiae* and *H. Sapiens* are obtained from HitPredict [31], and also exploited for preparing one of the two developed views. Reported results are compared with state-of-the-art gene selection algorithms, and supported through proper biological significance and a visualization tests.

A thorough analysis of the reported results proves that our proposed CMVMC-based gene selection technique not only reduces the gene-space up to a reasonable scale but also ensures higher accuracy in sample classification. According to the current state of this work, one or more *‘omics’* data can be further incorporated in designing a multi-view clustering algorithm to make it more efficient in finding groups of functionally similar genes. The authors are currently working in that direction.

## Data Availability

All data sets used in the work are publicly available and the source reference are given in main manuscript.

## Abbreviations

CMVMC: Consensus Multi-View Multi-objective Clustering
GO: Gene Ontology
PPIN: Protein-Protein Interaction Network
FuSim: Fused Similarity measure
CLARANS: Clustering Large Applications based upon RANdomized Search
UMC: Unsupervised Multi-objective Clustering
XB: Xie Beni
AI: Agreement Index
AMOSA: Archived Multi Objective Simulated annealing
CA: Classification Accuracy
DB index: Davies Bouldin index
MBPSO: Multi-objective Binary Particle Swarm Optimization
k-NN: k Nearest Neighbour
RF: Random Forest
NB: Naive Bayes
MLP: Multi-layer Perceptron
